# Supplementary material for: Aberrant Multimodal Connectivity Pattern Involved in Default Mode Network and Limbic Network in Amyotrophic Lateral Sclerosis
Source: Brain Sci. 2023 May 15;13(5):803. doi: 10.3390/brainsci13050803 (PMC10216573; doi:10.3390/brainsci13050803)
Supplement: Supplementary file 1 [file brainsci-13-00803-s001.zip › brainsci-2301411-supplementary.docx]

**Supplementary Materials**

We applied the linear discriminant analysis and the bagged trees to separately assess the classification performance. These two models were performed in MATLAB 2017b. The linear discriminant analysis, which is known as subspace discriminant, attempts to effectively discriminate a data set between multiple classes by using Bayes optimal solution to assign data points to appropriate classes based on log-likelihood ratio. The bagged trees use a method known as bagging or bootstrap aggregation. The method creates many trees and averages the results of many trees using different thresholds and values for classification and averages them to determine better decision boundaries and create better prediction models. In order to compare with previous SVM models, the features in current models are consistent with them included in SVM models.

Firstly, the linear discriminant analysis was performed based on the different SC-FC coupling measures. Based on the features of global-network SC-FC coupling, the linear discriminant analysis could distinguish the ALS patients from HC with an accuracy of 74.4%, a sensitivity of 61.11% and a specificity of 84% (Supplementary Figure S1).


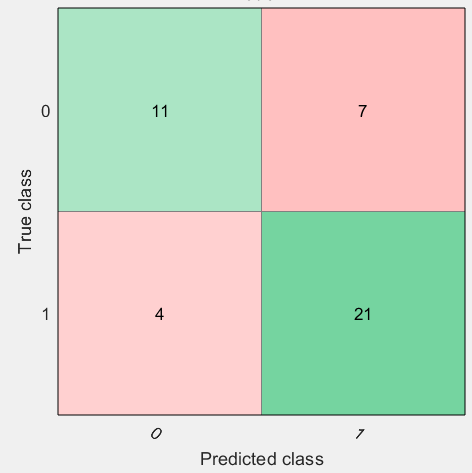

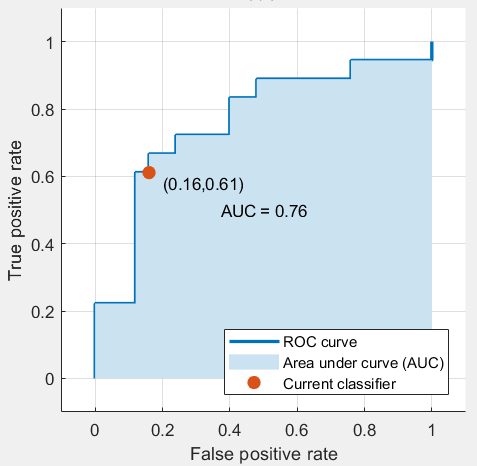


**Figure S1.** The global-network SC-FC coupling and linear discriminant analysis. 0 represents ALS and 1 represents HC. Abbreviations: AUC, area under curve; ALS, amyotrophic lateral sclerosis; HC, healthy controls.

By applying the regional-node SC-FC coupling as features, the linear discriminant analysis model showed the classification performance with an accuracy of 76.7%, a sensitivity of 66.67% and a specificity of 84% (Supplementary Figure S2).


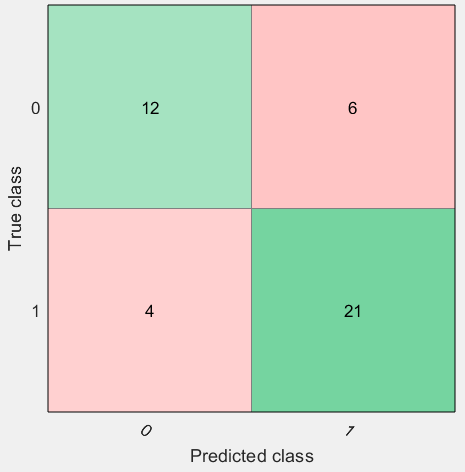

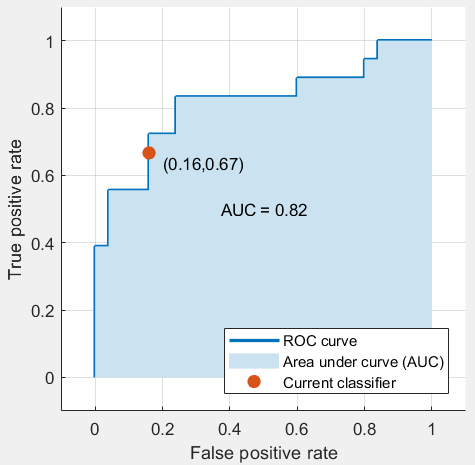


**Figure S2**. The regional-node SC-FC coupling and linear discriminant analysis. 0 represents ALS and 1 represents HC. Abbreviations: AUC, area under curve; ALS, amyotrophic lateral sclerosis; HC, healthy controls.

Based on the features of within-network SC-FC coupling, the linear discriminant analysis could distinguish the ALS patients from HC with an accuracy of 79.1%, a sensitivity of 77.78% and a specificity of 80% (Supplementary Figure S3).


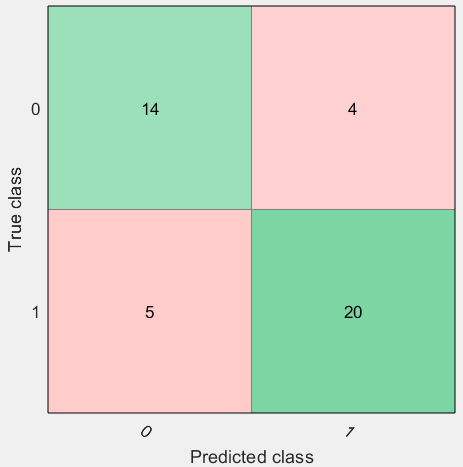

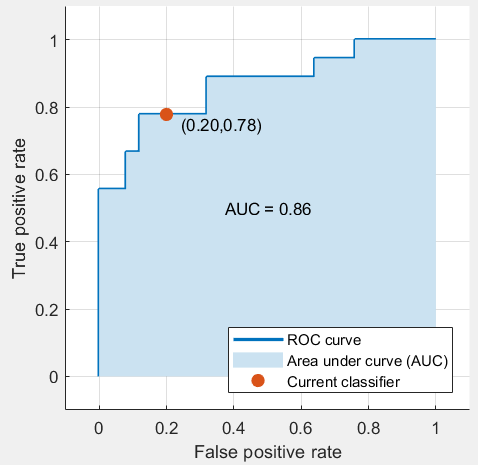


**Figure S3**. The within-network SC-FC coupling and linear discriminant analysis. 0 represents ALS and 1 represents HC. Abbreviations: AUC, area under curve; ALS, amyotrophic lateral sclerosis; HC, healthy controls.

By using the between-network SC-FC coupling as features, the linear discriminant analysis showed the classification performance with an accuracy of 69.8%, a sensitivity of 61.11% and a specificity of 76% (Supplementary Figure S4).


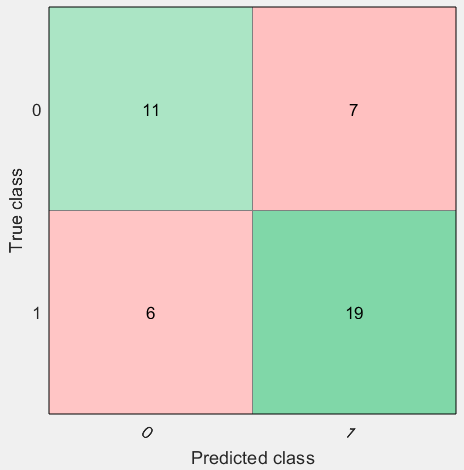

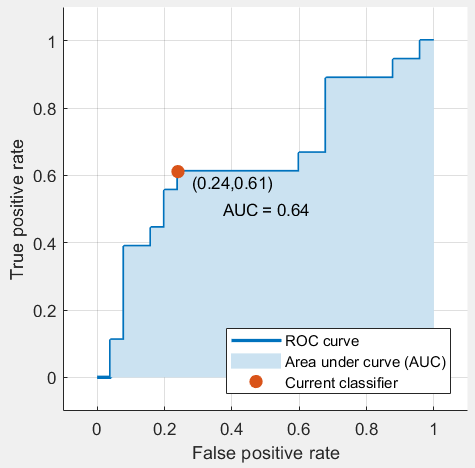


**Figure S4**. The between-network SC-FC coupling and linear discriminant analysis. 0 represents ALS and 1 represents HC. Abbreviations: AUC, area under curve; ALS, amyotrophic lateral sclerosis; HC, healthy controls.

Secondly, the bagged trees was performed based on the different SC-FC coupling measures. Based on the features of global-network SC-FC coupling, the bagged trees could distinguish the ALS patients from HC with an accuracy of 79.1%, a sensitivity of 77.78% and a specificity of 80% (Supplementary Figure S5).


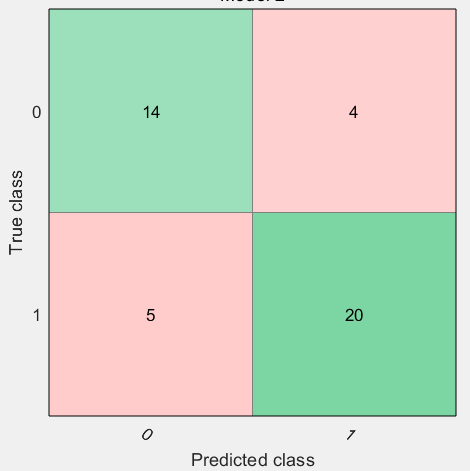

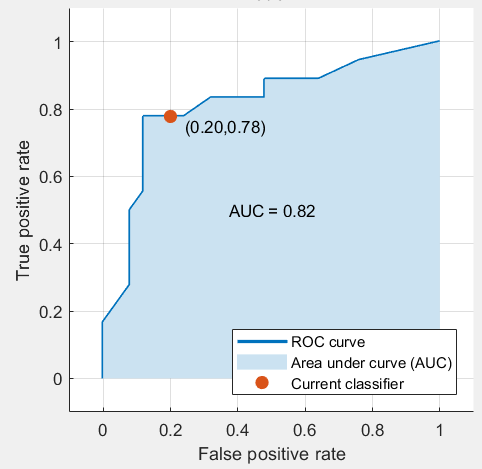


**Figure S5.** The global-network SC-FC coupling and bagged trees. 0 represents ALS and 1 represents HC. Abbreviations: AUC, area under curve; ALS, amyotrophic lateral sclerosis; HC, healthy controls.

By applying the regional-node SC-FC coupling as features, the bagged trees model showed the classification performance with an accuracy of 74.4%, a sensitivity of 61.11% and a specificity of 84% (Supplementary Figure S6).


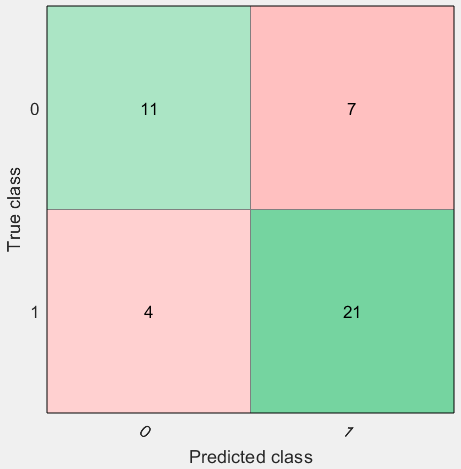

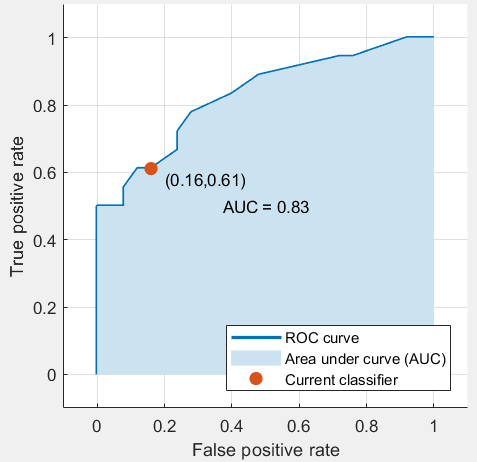


**Figure S6**. The regional-node SC-FC coupling and bagged trees. 0 represents ALS and 1 represents HC. Abbreviations: AUC, area under curve; ALS, amyotrophic lateral sclerosis; HC, healthy controls.

Based on the features of within-network SC-FC coupling, the bagged trees could distinguish the ALS patients from HC with an accuracy of 76.7%, a sensitivity of 72.22% and a specificity of 80% (Supplementary Figure S7).


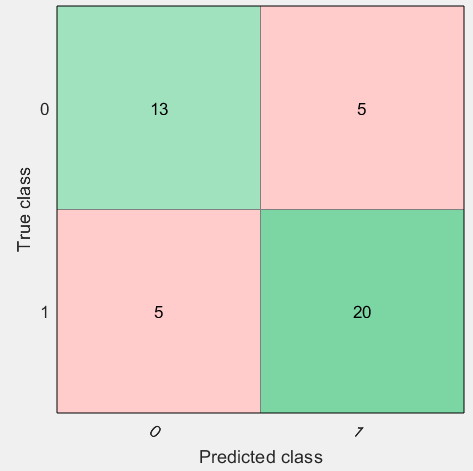

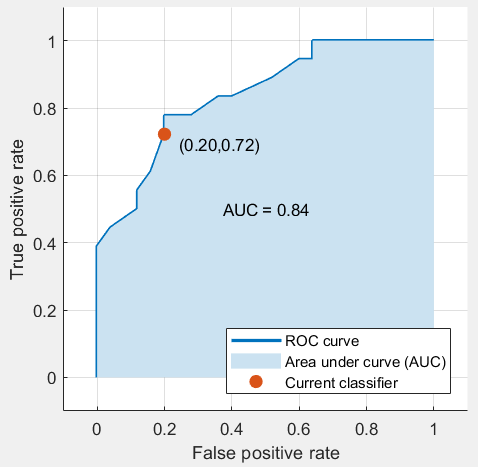


**Figure S7**. The within-network SC-FC coupling and bagged trees. 0 represents ALS and 1 represents HC. Abbreviations: AUC, area under curve; ALS, amyotrophic lateral sclerosis; HC, healthy controls.

By using the between-network SC-FC coupling as features, the bagged trees showed the classification performance with an accuracy of 74.4%, a sensitivity of 61.11% and a specificity of 84% (Supplementary Figure S8).


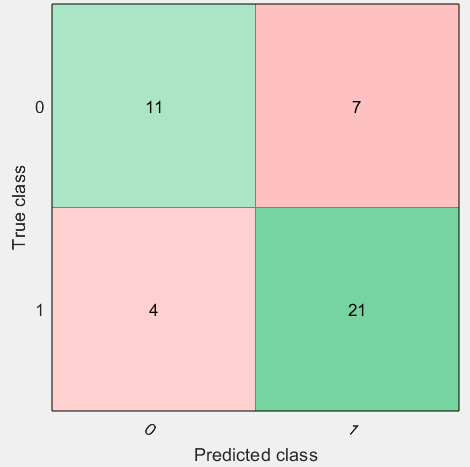

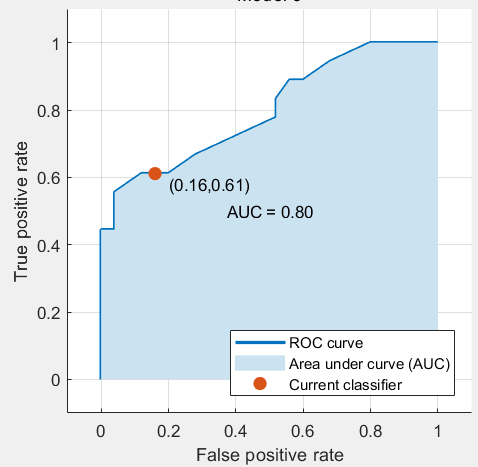


**Figure S8**. The between-network SC-FC coupling and bagged trees. 0 represents ALS and 1 represents HC. Abbreviations: AUC, area under curve; ALS, amyotrophic lateral sclerosis; HC, healthy controls.

**Table S1.** Brain areas and their abbreviations in Brainnetome Atlas.

| **Lobe** | **Gyrus** | **Left and Right Hemisphere** | **Lobe** | **Gyrus** | **Left and Right Hemisphere** |
| --- | --- | --- | --- | --- | --- |
| **Frontal Lobe** | SFG, Superior Frontal Gyrus | SFG_L(R)_7_1 | **Parietal Lobe** | SPL, Superior Parietal Lobule | SPL_L(R)_5_1 |
|  |  | SFG_L(R)_7_2 |  |  | SPL_L(R)_5_2 |
|  |  | SFG_L(R)_7_3 |  |  | SPL_L(R)_5_3 |
|  |  | SFG_L(R)_7_4 |  |  | SPL_L(R)_5_4 |
|  |  | SFG_L(R)_7_5 |  |  | SPL_L(R)_5_5 |
|  |  | SFG_L(R)_7_6 |  | IPL, Inferior Parietal Lobule | IPL_L(R)_6_1 |
|  |  | SFG_L(R)_7_7 |  |  | IPL_L(R)_6_2 |
|  | MFG, Middle Frontal Gyrus | MFG_L(R)_7_1 |  |  | IPL_L(R)_6_3 |
|  |  | MFG_L(R)_7_2 |  |  | IPL_L(R)_6_4 |
|  |  | MFG_L(R)_7_3 |  |  | IPL_L(R)_6_5 |
|  |  | MFG_L(R)_7_4 |  |  | IPL_L(R)_6_6 |
|  |  | MFG_L(R)_7_5 |  | Pcun, Precuneus | PCun_L(R)_4_1 |
|  |  | MFG_L(R)_7_6 |  |  | PCun_L(R)_4_2 |
|  |  | MFG_L(R)_7_7 |  |  | PCun_L(R)_4_3 |
|  | IFG, Inferior Frontal Gyrus | IFG_L(R)_6_1 |  |  | PCun_L(R)_4_4 |
|  |  | IFG_L(R)_6_2 |  | PoG, Postcentral Gyrus | PoG_L(R)_4_1 |
|  |  | IFG_L(R)_6_3 |  |  | PoG_L(R)_4_2 |
|  |  | IFG_L(R)_6_4 |  |  | PoG_L(R)_4_3 |
|  |  | IFG_L(R)_6_5 |  |  | PoG_L(R)_4_4 |
|  |  | IFG_L(R)_6_6 | **Insular Lobe** | INS, Insular Gyrus | INS_L(R)_6_1 |
|  | OrG, Orbital Gyrus | OrG_L(R)_6_1 |  |  | INS_L(R)_6_2 |
|  |  | OrG_L(R)_6_2 |  |  | INS_L(R)_6_3 |
|  |  | OrG_L(R)_6_3 |  |  | INS_L(R)_6_4 |
|  |  | OrG_L(R)_6_4 |  |  | INS_L(R)_6_5 |
|  |  | OrG_L(R)_6_5 |  |  | INS_L(R)_6_6 |
|  |  | OrG_L(R)_6_6 | **Limbic Lobe** | CG, Cingulate Gyrus | CG_L(R)_7_1 |
|  | PrG, Precentral Gyrus | PrG_L(R)_6_1 |  |  | CG_L(R)_7_2 |
|  |  | PrG_L(R)_6_2 |  |  | CG_L(R)_7_3 |
|  |  | PrG_L(R)_6_3 |  |  | CG_L(R)_7_4 |
|  |  | PrG_L(R)_6_4 |  |  | CG_L(R)_7_5 |
|  |  | PrG_L(R)_6_5 |  |  | CG_L(R)_7_6 |
|  |  | PrG_L(R)_6_6 |  |  | CG_L(R)_7_7 |
|  | PCL, Paracentral Lobule | PCL_L(R)_2_1 | **Occipital Lobe** | Cun, Cuneus Gyrus | Cun_L(R)_5_1 |
|  |  | PCL_L(R)_2_2 |  |  | Cun _L(R)_5_2 |
| **Temporal Lobe** | STG, Superior Temporal Gyrus | STG_L(R)_6_1 |  |  | Cun _L(R)_5_3 |
|  |  | STG_L(R)_6_2 |  |  | Cun _L(R)_5_4 |
|  |  | STG_L(R)_6_3 |  |  | Cun _L(R)_5_5 |
|  |  | STG_L(R)_6_4 |  | OcG, Occipital Gyrus | OcG_L(R)_4_1 |
|  |  | STG_L(R)_6_5 |  |  | OcG _L(R)_4_2 |
|  |  | STG_L(R)_6_6 |  |  | OcG _L(R)_4_3 |
|  | MTG, Middle Temporal Gyrus | MTG_L(R)_4_1 |  |  | OcG_L(R)_4_4 |
|  |  | MTG_L(R)_4_2 |  | sOcG, Superior Occipital Gyrus | sOcG _L(R)_2_1 |
|  |  | MTG_L(R)_4_3 |  |  | sOcG _L(R)_2_2 |
|  |  | MTG_L(R)_4_4 | **Subcortical Nuclei** | Amyg, Amygdala | Amyg_L(R)_2_1 |
|  | ITG, Inferior Temporal Gyrus | ITG_L(R)_7_1 |  |  | Amyg_L(R)_2_2 |
|  |  | ITG_L(R)_7_2 |  | Hipp, Hippocampus | Hipp_L(R)_2_1 |
|  |  | ITG_L(R)_7_3 |  |  | Hipp_L(R)_2_2 |
|  |  | ITG_L(R)_7_4 |  | Str, Striatum | Str_L(R)_6_1 |
|  |  | ITG_L(R)_7_5 |  |  | Str_L(R)_6_2 |
|  |  | ITG_L(R)_7_6 |  |  | Str_L(R)_6_3 |
|  |  | ITG_L(R)_7_7 |  |  | Str_L(R)_6_4 |
|  | FuG, Fusiform Gyrus | FuG_L(R)_3_1 |  |  | Str_L(R)_6_5 |
|  |  | FuG_L(R)_3_2 |  |  | Str_L(R)_6_6 |
|  |  | FuG_L(R)_3_3 |  | Tha, Thalamus | Tha_L(R)_8_1 |
|  | PhG, Parahippocampal Gyrus | PhG_L(R)_6_1 |  |  | Tha_L(R)_8_2 |
|  |  | PhG_L(R)_6_2 |  |  | Tha_L(R)_8_3 |
|  |  | PhG_L(R)_6_3 |  |  | Tha_L(R)_8_4 |
|  |  | PhG_L(R)_6_4 |  |  | Tha_L(R)_8_5 |
|  |  | PhG_L(R)_6_5 |  |  | Tha_L(R)_8_6 |
|  |  | PhG_L(R)_6_6 |  |  | Tha_L(R)_8_7 |
|  | pSTS, posterior Superior Temporal Sulcus | pSTS_L(R)_2_1 |  |  | Tha_L(R)_8_8 |
|  |  | pSTS_L(R)_2_2 |  | | |
